# S03-4 3PL: Promoting pupils’ physical literacy: a pilot study testing feasibility and acceptability of the Y-PATH intervention in a Danish school setting

**DOI:** 10.1093/eurpub/ckac093.015

**Published:** 2022-08-29

**Authors:** Mette Kurtzhals, Paulina Sander Melby, Peter Elsborg, Glen Nielsen, Sarahjane Belton, Johann Issartel, Wesley O’Brien, Peter Bentsen

**Affiliations:** 1 Center for Clinical Research and Prevention, Copenhagen University Hospital – Bisbebjerg and Frederiksberg, Frederiksberg, Denmark; 2 Department of Nutrition, Exercise and Sports, University of Copenhagen, Copenhagen, Denmark; 3 Danish School sports; 4 School of Health and Human Performance, Dublin City University, Dublin, Ireland; 5 School of Education, University College Cork (UCC), Ireland; 6 Department of Geosciences and Natural Resource Management, University of Copenhagen, Copenhagen, Denmark

**Keywords:** Health promotion, intervention, physical literacy, school

## Abstract

**Background:**

A considerable number of Danish children and adolescents do not currently meet the national physical activity (PA) recommendations. In recent years, the concept of physical literacy (PL), has gained popularity worldwide, and it is considered as a proximal measure for lifelong PA. However, only a few interventions targeting PL exist on a global scale. In Denmark, the development of theoretically driven and evidence-based PL interventions that aim to increase PL among children and adolescents has not begun. Yet, a promising, theory-based, and internationally tested intervention, the Youth Physical Activity Towards Health (Y-PATH), has proven to be effective on children and adolescents’ PA levels and motor skills. This presentation introduces the Promoting Pupils’ Physical Literacy (3PL) project which aims to test the feasibility and acceptability of the Y-PATH intervention in a Danish context among pupils 9-11 years of age.

**Methods and Results:**

The hypothesis is that a revised and adapted 3PL intervention protocol that aims to increase pupils’ PL is ready for effectiveness testing by the end of this project. Two public schools will be recruited and randomly assigned to intervention or control condition following a waitlist design. The primary outcomes include the feasibility and acceptability of the intervention. The feasibility of the practicality and the recruitment process will be assessed within a document log administered by research assistants. The acceptability, including demand and experiences, and the intervention implementation degree will be evaluated by short bimonthly questionnaires to teachers, and interviews with pupils, teachers, parents, and school managers. The preliminary effectiveness will be tested by comparing changes in pupils’ PL over time, assessed with the validated Danish Assessment of Physical Literacy tool.

**Discussion:**

A revised PL intervention, a Template for Intervention Description and Replication TIDieR checklist, and a protocol that offers a solid empirical and theoretical foundation for a future upscaled effectiveness study. Additionally, the development of such protocol and checklist provides a national as well as an international opportunity for researchers to use and compare effectiveness of the intervention across countries.

